# Cortical activity evoked by an acute painful tissue-damaging stimulus in healthy adult volunteers

**DOI:** 10.1152/jn.00990.2012

**Published:** 2013-02-20

**Authors:** Lorenzo Fabrizi, Gemma Williams, Amy Lee, Judith Meek, Rebeccah Slater, Sofia Olhede, Maria Fitzgerald

**Affiliations:** ^1^Department of Neuroscience, Physiology and Pharmacology, University College London, London, United Kingdom;; ^2^Department of Statistical Science, University College London, London, United Kingdom; and; ^3^Elizabeth Garrett Anderson Obstetric Wing, University College Hospital, London, United Kingdom

**Keywords:** pain, nociception, cortex, brain, event-related potential

## Abstract

Everyday painful experiences are usually single events accompanied by tissue damage, and yet most experimental studies of cutaneous nociceptive processing in the brain use repeated laser, thermal, or electrical stimulations that do not damage the skin. In this study the nociceptive activity in the brain evoked by tissue-damaging skin lance was analyzed with electroencephalography (EEG) in 20 healthy adult volunteers (13 men and 7 women) aged 21–40 yr. Time-frequency analysis of the evoked activity revealed a distinct late event-related vertex potential (lance event-related potential, LERP) at 100–300 ms consisting of a phase-locked energy increase between 1 and 20 Hz (delta-beta bands). A pairwise comparison between lance and sham control stimulation also revealed a period of ultralate stronger desynchronization after lance in the delta band (1–5 Hz). Skin application of mustard oil before lancing, which sensitizes a subpopulation of nociceptors expressing the cation channel TRPA1, did not affect the ultralate desynchronization but reduced the phase-locked energy increase in delta and beta bands, suggesting a central interaction between different modalities of nociceptive inputs. Verbal descriptor screening of individual pain experience revealed that lance pain is predominantly due to Aδ fiber activation, but when individuals describe lances as C fiber mediated, an ultralate delta band event-related desynchronization occurs in the brain-evoked activity. We conclude that pain evoked by acute tissue damage is associated with distinct Aδ and C fiber-mediated patterns of synchronization and desynchronization of EEG oscillations in the brain.

electroencephalographic (EEG) brain activity in response to noxious experimental stimulation has been extensively studied in human adult volunteers. The versatility of stimulation techniques such as cutaneous laser, mechanical, electrical, and thermal stimulation has allowed characterization and differentiation of the components of the event-related potentials (ERPs) elicited by different peripheral noxious modalities. After peripheral stimulation, evoked responses to Aδ and C nociceptive afferent inputs to the brain can be detected as long-latency (100–400 ms) and ultralong-latency (800–1,500 ms) neuronal activity in EEG traces (Kakigi et al. 2000), depending upon the site and modality of stimulation. A late potential, consisting of a negative and a positive deflection maximal at the vertex, ascribed to activation of Aδ afferents, has been reported after a wide variety of noxious stimulation, such as electrical (Bromm and Scharein 1982; Miltner et al. 1989; Naka and Kakigi 1998), mechanical (Bromm and Scharein 1982), contact heat (Chao et al. 2008; Chen et al. 2001; Harkins et al. 2000), and laser radiant heat (Bromm and Treede 1987; Mouraux et al. 2003; Zhang et al. 2012). This ERP, which differs in latency and in labeling according to the stimulus modality, has been most commonly studied in response to laser radiant heat, when it is often termed laser-evoked potential (LEP). The amplitude of the LEP has been found to correlate partially with subjective pain report (Chen et al. 1998; Garcia-Larrea et al. 1997; Iannetti et al. 2004; Kakigi et al. 1989) and pain awareness (Valls-Sole et al. 2012), but a dissociation with subject pain report has also been reported, consistent with evidence that the LEP reflects stimulus saliency rather than pain intensity (Iannetti et al. 2008; Legrain et al. 2002; Mouraux and Iannetti 2008).

Recent studies of nociceptive EEG responses have highlighted the importance of analyzing not only phase-locked ERPs but also events that are time-locked but not phase-locked to the stimulation and are therefore lost in the average in the time domain. Thus event-related synchronization (ERS) and event-related desynchronization (ERD) in specific frequency bands have been observed in response to laser stimulation (Domnick et al. 2009; Mouraux et al. 2003; Zhang et al. 2012). These observations may provide more information about brain activity in relation to afferent input and perceived pain intensity than the phase-locked evoked potentials alone. Selective activation of peripheral sensory C fibers with laser stimulation combined with an A-fiber block, low-intensity or small-area stimulation (Bromm and Treede 1987; Mouraux et al. 2003) evokes an ultralate positive ERP, corresponding to an ultralate phase-locked energy increase in the delta frequency band. Even without special stimulation techniques and therefore in the absence of the ultralate ERP, C-fiber input elicits non-phase-locked alpha ERD and beta ERS (Domnick et al. 2009l Mouraux et al. 2003). More recently, non-phase-locked gamma ERD has been shown to correlate well with pain intensity independent of saliency (Zhang et al. 2012). In addition, acute and chronic topical application of irritant chemicals, such as capsaicin and mustard oil, which stimulate subpopulations of small- and medium-diameter nociceptors, have been shown to alter the activity evoked by noxious stimuli in the time and frequency domains (Beydoun et al. 1996; Domnick et al. 2009), reflecting changes in perception and hyperalgesia (Koltzenburg et al. 1992). The Aδ and C nociceptor afferent volleys and their subsequent processing are believed to respectively underlie the perception of first pain, described as well localized, sharp, and pricking, and second pain, described as diffuse and burning (Bromm and Treede 1984). These properties, together with the selective activation of the two fiber types by different stimulus modalities, have been used to devise a novel assessment tool that discriminates between Aδ and C fiber-mediated pain based on the selection of verbal descriptors from the McGill Pain Questionnaire (Beissner et al. 2010).

These pioneering studies of experimental pain in healthy volunteers have focused on noxious stimuli that avoid tissue damage. This method allows repeated stimulation in the same session, thereby maximizing signal-to-noise ratio. However, many painful experiences in real life are associated with tissue damage. A skin-breaking stimulus, such as a lance, provides a suitable model for these kind of events that cannot otherwise be obtained with a laser beam or an electrical stimulus, albeit less selective for Aδ or C fiber nociceptors. However, to date, the brain response to a skin-breaking stimulus remains unknown. In this study, we have characterized, for the first time, the cortical activity evoked by a noxious cutaneous tissue-damaging stimulation. To do this we recorded the EEG activity time-locked to finger lances in naive human adults. This is a frequently required procedure for clinical care, but also a model of accidental tissue damage. In particular, we investigated the relation of the evoked activity to the peripheral sensory input and the differential role of Aδ and C nociceptors in its generation. First, we compared the response to the lance to a similar non-tissue-damaging stimulation so that we could identify which components of the response were specifically associated to the tissue damage. Subsequently, in order to identify the cortical correlates of peripheral Aδ and C nociceptor activations, we *1*) assessed the effect of local sensitization of C fibers expressing the transient receptor potential ankyrin-1 (TRPA1+) by comparing the response to a lance on a finger treated with mustard oil with that to a lance on a finger treated with an inactive compound and *2*) compared responses classified as Aδ or C fiber mediated according to a three-verbal descriptors discriminator. This study provides an insight into the underlying brain dynamics that generate acute pain, in particular those that mediate cortical affective information regarding real tissue-damaging events that threaten the integrity of the body.

## METHODS

### Participants

Twenty healthy adult volunteers (13 men and 7 women) aged 21–40 yr (28.6 ± 6.5 yr, mean ± SD) participated in this study. All participants gave their written informed consent. The study conformed to the standards set by the Declaration of Helsinki and was approved by the University College London Research Ethics committee.

### EEG Recording

Recording electrodes (disposable Ag/AgCl cup electrodes) were positioned according to the modified international 10/20 electrode placement system at F7, F8, Cz, CPz, C3, C4, CP3, CP4, T3, T4, T5, T6, O1, and O2. Reference and ground electrodes were placed at FCz and the chest, respectively. EEG activity, from DC to 70 Hz, was recorded with the Neuroscan SynAmps2 EEG/EP recording system. Signals were digitized with a sampling rate of 2 kHz and a resolution of 24 bits.

### Cutaneous Stimuli

The noxious cutaneous tissue-damaging procedure was a lance performed on the palmar surface of the distal phalanx of the fifth finger of each hand with a sterile lancet (Tenderfoot, International Technidyne). The lancet houses a 2.5-mm spring-loaded blade, clinically used to incise the skin for blood sampling. When the device is activated by pressing a trigger on its superior surface, the blade swings in an arc such that for a short time the blade protrudes from the device and makes an incision to a depth of 1 mm in the superficial layers of the skin. After the blade is released it automatically and permanently retracts. The release of the blade produced a single event mark on the EEG recording using an accelerometer mounted to the superior surface of the lancet that detected the vibration caused by the event (Worley et al. 2012). A sham control non-tissue-damaging procedure was performed by rotating the lancet by 90° so that the blade did not enter the skin.

### Peripheral Sensitization

To test the influence of peripheral sensitization upon the lance response, either 100 μl of mustard oil (95% allyl isothiocyanate; Sigma-Aldrich) or 100 μl of inactive mineral oil (Sigma-Aldrich) was applied on either finger. The participants were blind to the side of mustard oil application. Quantitative sensory testing (QST) was conducted to assess the effect of mustard oil on mechanical and pressure pain threshold with PinPrick stimulators (MRC Systems) and a pressure algometer. Three PinPrick stimulators, small-diameter calibrated punctate rods, were used: 128, 256, and 512 mN. All stimuli were applied twice and in random order, and the participants were blind to the force used. The participants were asked to score each pinprick independently on a scale of 0–100, where 0 was “no pain” and 100 was their “worst pain imaginable.” The pressure algometer, with a surface area of 7.1 mm^2^ eliciting a constant force of 66 g (9.3 g/mm^2^), was used to assess pressure pain. The algometer was placed just below the nail bed on the fifth fingers and removed at the time at which the participants reported feeling pain. This time was recorded as a measure of pressure pain threshold.

### Pain Report

After each lance, participants were asked to score the pain on a scale of 0–100, where 0 indicated “no pain” and 100 “worst pain imaginable.” Subjects were then asked to describe the quality of the pain by choosing as many words as they wanted from a list of 67 descriptors from the McGill Pain Questionnaire (Melzack 1975). The results were used to characterize the pain sensation evoked by the lance as transmitted via Aδ or C fibers with a validated verbal discriminator (Beissner et al. 2010).

### Experimental Protocol

Studies were conducted in a quiet, temperature-controlled room purpose-built for research and lasted a maximum of 1 h, with an average time of 45 min. Experimental preparation involved EEG setup and application of compounds. Participants were seated while a qualified clinical physiologist placed the scalp electrodes for EEG recordings. Electrode/skin contact impedance was kept to a minimum by abrading the skin with EEG prepping gel and using EEG conductive paste. Electrodes were held in place with an elastic net, and leads were tied together to minimize electrical interference. Participants were blind to the side of mustard oil application; they were asked to close their eyes and keep their hands at waist level. Mustard oil and inactive mineral oil were applied by placing 1-cm^2^ gauzes soaked with 100 μl of either compound on the palmar surface of the distal phalanx of the fifth finger of either hand. In half of the participants mustard oil was applied on the right hand and in the other half on the left hand. The fingers were then covered with an occlusion dressing for 5 min before the skin was cleansed with antiseptic. Participants were then instructed to keep their eyes closed and their hands with the palms facing up to conduct QST.

Participants then lay supine on a hospital bed for EEG recording. They were asked to relax their jaw (to reduce muscle artifact) and close their eyes (to reduce eyeblink artifact) while counting backward (to reduce alpha activity). Once the EEG activity had stabilized, the sham control and the two lances were conducted sequentially, leaving >1 min between stimuli. The sham control stimulation was always performed prior to the lances, the first of which was conducted on the right hand in half of the participants and on the left hand in the other half. Participants were informed that they would receive a sham control or a noxious stimulation but were unaware of when the stimuli would occur. Skin wounds were wiped with antiseptic and dressed with cotton wool. Pain scoring was reported after each lance, while the pain description questionnaire was filled in at the end of the experiment for each lance independently.

### EEG Analysis

#### Preprocessing.

EEG data were segmented into epochs of 6 s, from 3 s before stimulus to 3 s after stimulus. Each epoch was baseline corrected with the prestimulus interval from −3 s to 0 s as a reference. Eyeblinks and movements were removed from contaminated trials in EEGLAB with independent component analysis (ICA) and the CORRMAP toolbox (Delorme and Makeig 2004; Viola et al. 2009). Independent components clearly representing eyeblinks were low-pass filtered at 20 Hz with a zero-phase 2nd-order Butterworth filter and then removed from the traces. Two lance EEG epochs from the same subject and one control epoch from another subject were rejected after visual inspection because of contamination by muscle or gross movement artifacts.

Individual trials were then grouped into *1*) lances with inactive compound, *2*) lances with mustard oil, and *3*) sham controls. Subsequently, all lance trials were also grouped according to verbal description into events that fitted the Aδ fiber- or C fiber-mediated pain definition or did not fit into either of these two classifications.

#### ERP analysis.

EEG data were band-pass filtered between 1 and 30 Hz with a zero-phase 2nd-order Butterworth filter and resegmented into epochs of 1.5 s, from 0.5 s before stimulus to 1 s after stimulus. Each epoch was baseline corrected with the prestimulus interval −0.5 s to 0 s as a reference. N and P peaks at the vertex (Cz) were identified in individual traces by a qualified clinical physiologist. This was done by comparing the individual traces with the grand average across all subjects and selecting the peak that most resembled those of the average. The amplitudes and the latencies of the N and P waves were compared between lances and sham controls and lances on fingers treated with inactive compound or mustard oil with a two-tailed Student's paired *t*-test. Scalp topography maps were obtained from the group averages, every 10 ms in 40-ms intervals, centered on the most negative and most positive deflection following stimulation. Comparison by verbal descriptors was conducted with a one-way ANOVA, and in case of rejection of the null hypothesis, we performed a post hoc analysis using a Student's *t*-test to compare the responses belonging to the Aδ, C, and no fiber categories.

#### Time-frequency analysis.

EEG data were high-pass filtered at 1 Hz with a zero-phase 2nd-order Butterworth filter. Time-frequency analysis (TF) was performed with a complex Morse wavelet transform (Olhede and Walden 2002) for the vertex electrode (Cz). This allowed us to calculate a complex time-frequency (i.e., wavelet-scale) spectral estimate *W*(*a*,*b*) of the EEG signal at each point (*a*,*b*) of the time-frequency plane from 3 s before stimulus to 3 s after stimulus in the time domain and between 1 and 70 Hz (in logarithmic steps) in the frequency domain. We estimated the stimulus-induced energy changes time-locked to the lances on fingers treated with inactive compound, to the sham controls, and to the lances on fingers treated with mustard oil. We then compared these patterns of evoked activity between lances and sham controls and between lances on fingers with inactive compound or mustard oil. Subsequently, we regrouped the lances according to verbal description and compared the patterns of evoked activity associated with lances described as mediated by Aδ, C, or no specific fibers.

The time-frequency spectral energy changes in the EEG that were induced by the stimuli were estimated on a group average basis because only one trial per condition per participant was available. This was done in two ways: *1*) by calculating the energy (i.e., modulus square) of the TF transform for each individual trial and then averaging them in the time-frequency domain (“TF-single”) and *2*) by averaging the trials in the time domain and then calculating the energy of the TF transform of the resulting average (“TF-group”) as described in the appendix. To compare the patterns of evoked activity between different groups we conducted pairwise comparisons when possible or group comparisons otherwise (appendix). “TF-single” represents evoked EEG activity that is phase-locked or non-phase-locked to the stimulation, while “TF-group” represents only EEG activity that is phase-locked in the same way for all participants/trials—i.e., whose phase initialization is coherent across participants/trials (Mouraux et al. 2003). Therefore, EEG changes present in both representations should be considered as being phase-locked across participants/trials, while those that are only present in TF-single should be considered as non-phase-locked.

The significance level was assumed to be 0.05 for all tests; however, because these tests were conducted at any point (*a*,*b*) of the time-frequency plane, the false discovery rate was used to correct for multiple comparisons (Benjamini and Hochberg 1995). The total number of independent tests performed was estimated in two steps: *1*) calculating the number of independent tests at each frequency by diving the length of the considered epoch by the length of the wavelet at that frequency (accounting for the correlation caused by the smoothing in time of the wavelet transform) and *2*) adding those numbers across frequencies.

## RESULTS

### Adult Response to Noxious Cutaneous Tissue Damage

**Fig. 1.**
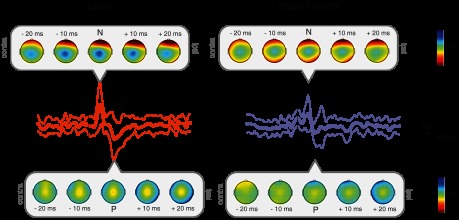
Grand average (±SD) event-related potential (ERP) waveforms in response to a finger lance (*n* = 19) and sham control (*n* = 19) at Cz, with topography maps at N and P and at 20 ms and 10 ms before and after the peaks.

Finger lances were considered painful by all participants, with an average pain score of 40.0 ± 23.4 (range: 8–90), and evoked a distinct pattern of brain activity. Both lance and sham control stimulation evoked clear ERPs, consisting of a negative and a positive peak, which differed in amplitude and latency (Fig. 1). Lancing the fingers evoked a lance event-related potential (LERP) with mean N and P waves −10.78 ± 5.82 μV and 9.07 ± 5.21 μV in amplitude and 130 ± 40 ms and 258 ± 61 ms in latency, respectively (*n* = 19) (Table 1). In comparison, the N and P waves evoked by the sham control stimulation were −6.67 ± 5.87 μV and 5.12 ± 4.81 μV in amplitude and 103 ± 33 ms and 178 ± 36 ms in latency, respectively (*n* = 19) (Table 1). The peak-to-peak amplitude of the NP complex was significantly larger after the lance (19.85 ± 9.45 μV) compared with the control sham (11.79 ± 9.11 μV; paired *t*-test *P* = 0.02). When the N and P peaks were investigated separately, differences between control sham and lance were only significant for P amplitude (paired *t*-test: N, *P* = 0.054; P, *P* = 0.043) and latency (paired *t*-test: N, *P* = 0.057; P, *P* < 0.001).

**Table 1.** Peak latency and amplitude and peak-to-peak amplitude of the event-related potentials

	Lance		
	Inactive compound	Mustard oil	Control Sham
N			
Amplitude, μV	−10.78 ± 5.82	−8.63 ± 4.34	−6.67 ± 5.87
Latency, ms	130 ± 40	138 ± 41	103 ± 33
P			
Amplitude, μV	9.07 ± 5.21	7.59 ± 4.65	5.12 ± 4.81
Latency, ms	258 ± 61	240 ± 45	178 ± 36
Peak to peak, μV	19.85 ± 9.45	16.22 ± 8.44	11.79 ± 9.11

Values are mean ± SD latency at the peak and N and P components' and peak-to-peak amplitude of the event-related potential after lance in the presence of inactive compound or mustard oil and after control sham stimulation.

**Fig. 2.**
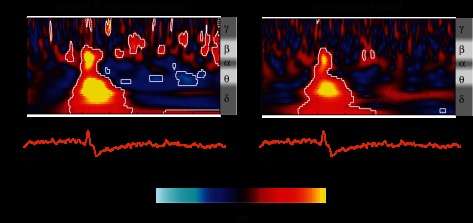
Time-frequency decomposition of the EEG responses to finger lances at Cz. *Left*: average of the time-frequency transforms of single trials (TF-single) revealing both phase-locked and non-phase-locked energy changes related to the stimulation. *Right*: time-frequency transform of the grand average of the trials (TF-group) revealing only stimulus-related changes phase-locked across subjects. Results are displayed as increases and decreases of energy (probability cumulative density function, CDF) relative to baseline (−1,000 to −500 ms). Values between 0 and 0.5 correspond to energy decreases, while values between 0.5 and 1 correspond to energy increases. Circumscribed areas represent significant energy changes relative to baseline. An approximated EEG frequency band division is displayed next to each time-frequency plot.

**Fig. 3.**
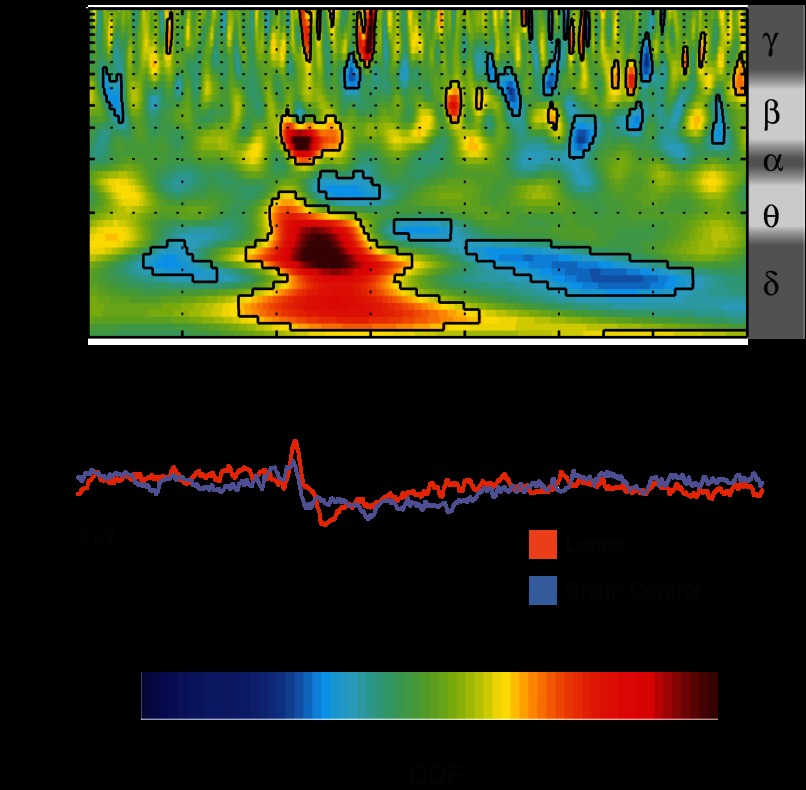
Pairwise comparison of the time-frequency decomposition between lance and sham control at Cz. This comparison displays the difference in the phase-locked and non-phase-locked evoked activity between lance and sham control. Values between 0 and 0.5 correspond to lower energy after lance, while values between 0.5 and 1 correspond to higher energy after lance (compared with sham control). Circumscribed areas represent significant evoked activity differences between lance and sham control. An approximated EEG frequency band division is displayed next to the time-frequency plot.

Time-frequency analysis revealed event-related activity patterns. Such patterns were not all phase-locked to the stimuli and therefore not discernible on the time average (Fig. 2). Lance evoked a late phase-locked energy increase between 0 and 500 ms spanning from the delta to the beta frequency band (1–20 Hz), consistent with the ERP seen in the time average (Fig. 2). A pairwise comparison showed that this energy increase is significantly greater after lance compared with sham control, especially in the delta/theta (1–5 Hz) and alpha/beta (10–20 Hz) frequency bands, corresponding to the difference in the ERPs following the two stimuli (Fig. 3). Time-frequency analysis also showed a prolonged period of ultralate beta/gamma (15–40 Hz) ERS and of theta (3–10 Hz) ERD after lance, which could not be seen in the initial ERP analysis (Fig. 2). A pairwise comparison did not show a consistent difference between lance and sham control stimuli at these frequencies but revealed a period of stronger ultralate desynchronization after lance in the delta band (1–5 Hz) relative to the sham control stimulation (Fig. 3).

### Effect of Peripheral Sensitization

**Fig. 4.**
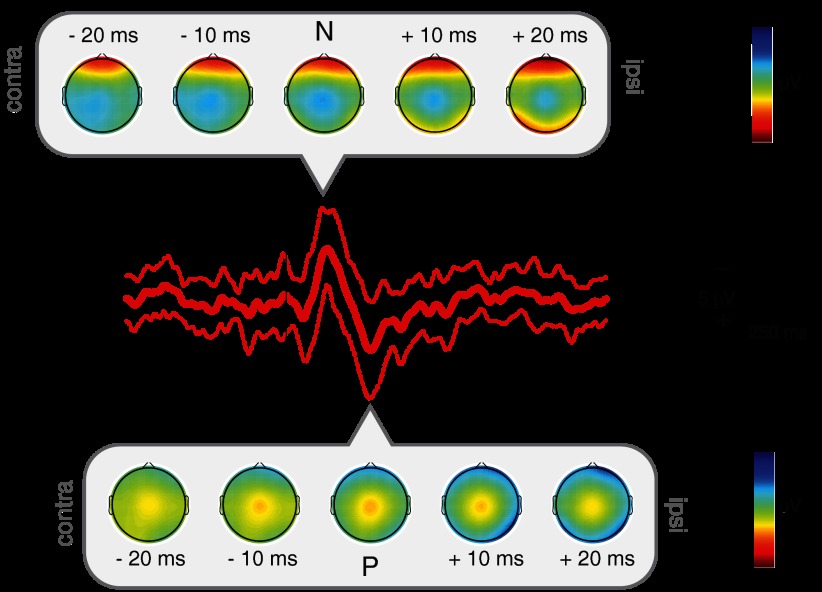
Grand average (±SD) ERP waveforms in response to lances on fingers treated with mustard oil at Cz (*n* = 19), with topography maps at N and P and at 20 ms and 10 ms before and after the peaks.

Mustard oil application to the finger was effective in causing sensitization to dynamic mechanical and pressure pain when tested with PinPrick stimulators of three intensities (128, 256, and 512 mN) and a pressure algometer (9.3 g/mm^2^). Mean pain ratings were significantly higher when the pinprick stimuli were applied on the finger treated with mustard oil (paired *t*-test: *P* < 0.001 for all stimulus intensities), and the latency to pain report on application of the pressure algometer was significantly shorter (paired *t*-test: *P* = 0.016) (Table 2). Despite this background sensitization, the pain rating given to lances in the presence of mustard oil (44.6 ± 27.5; range: 10–85) was not significantly different from inactive compound (40.0 ± 23.4; range: 8–90; paired *t*-test: *P* = 0.14). In agreement with this, the LERP was not affected by mustard oil (Fig. 4, Table 1), either in amplitude (paired *t*-test: N, *P* = 0.14; P, *P* = 0.18; peak-to-peak, *P* = 0.09) or in latency (paired *t*-test: N, *P* = 0.51; P, *P* = 0.18).

**Table 2.** Quantitative sensory testing in presence of inactive compound or mustard oil

	Inactive Compound	Mustard Oil
PinPrick		
128 mN	2.2 ± 2.7 (0–30)	6.6 ± 11.2 (0–40)
256 mN	12.1 ± 18.7 (0–90)	25 ± 23.9 (0–95)
512 mN	29.3 ± 27.3 (0–97)	37.4 ± 28.6 (0–100)
Algometer	64.2 ± 51.3 (8–180)	41.3 ± 62.2 (3–180)

Values are mean ± SD (range) pain ratings (on a 0–100 pain scale) in response to PinPricks of different weights in the presence of inactive compound or mustard oil and latency to pain report (in s) on application of the pressure algometer.

**Fig. 5.**
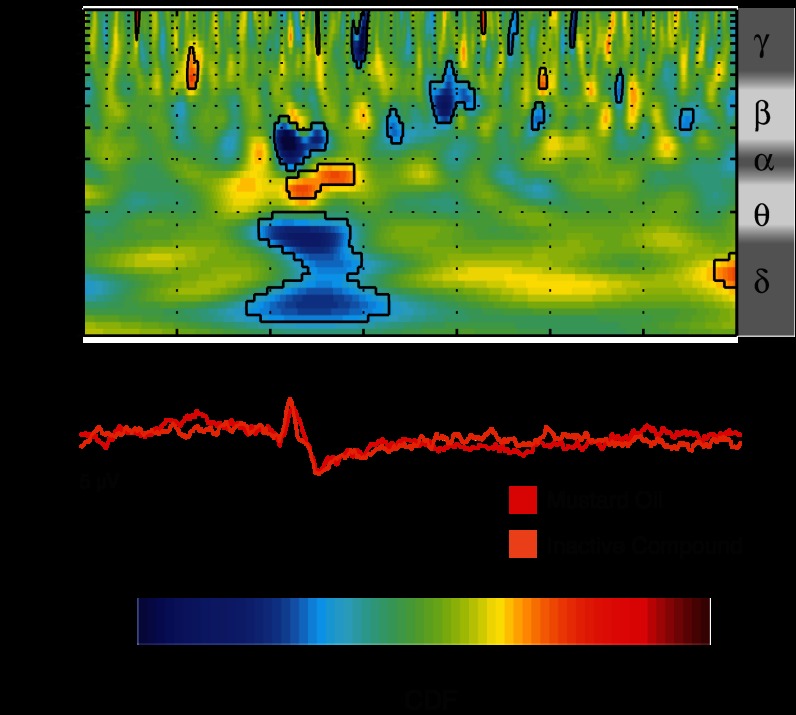
Pairwise comparison of time-frequency decompositions between lance on fingers with mustard oil and those with inactive compound at Cz. This comparison displays the difference in the phase-locked and non-phase-locked evoked activity. Values between 0 and 0.5 correspond to lower energy after lance on fingers with mustard oil, while values between 0.5 and 1 correspond to higher energy after lance on fingers with mustard oil (compared with inactive compound). Circumscribed areas represent significant evoked activity differences between lance on fingers with mustard oil and with inactive compound. An approximated EEG frequency band division is displayed next to the time-frequency plot.

However, a pairwise comparison of the time-frequency decompositions showed that the presence of mustard oil influences the late phase-locked event-related activity pattern corresponding to the LERP. This energy increase is significantly lower after lance on fingers treated with mustard oil, especially in the delta/theta (1–5 Hz) and alpha/beta (10–15 Hz) frequency bands, while greater in the theta/alpha (5–10 Hz) band (Fig. 5). There is no effect of mustard oil on the ultralate non-phase-locked energy changes.

### Nociceptive Fiber Components of the LERP Using Verbal Discrimination

**Fig. 6.**
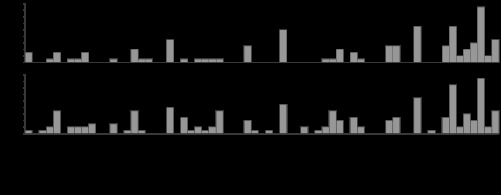
Descriptive words chosen to describe the pain sensation following finger lance.

The pain caused by the finger lance was defined according to a set of verbal descriptors that distinguish Aδ and C fiber-mediated pain (Beissner et al. 2010). The overall choice of descriptors after finger lance spanned a wide range of possible words but suggested more Aδ than C fibers (Fig. 6). Thus verbal descriptors from 20 lances (10 on fingers with inactive compound + 10 on fingers with mustard oil from 10 participants) implied Aδ fiber pain, while those from only 4 lances (3 on fingers with inactive compound + 1 on fingers with mustard oil from 4 participants) implied C fiber-mediated pain. The remaining 14 trials (6 on fingers with inactive compound + 8 on fingers with mustard oil from 9 participants) did not fit into either of these two classifications. Pain rating was not dependent on the verbal discrimination categories (1-way ANOVA: *P* = 0.769).

**Fig. 7.**
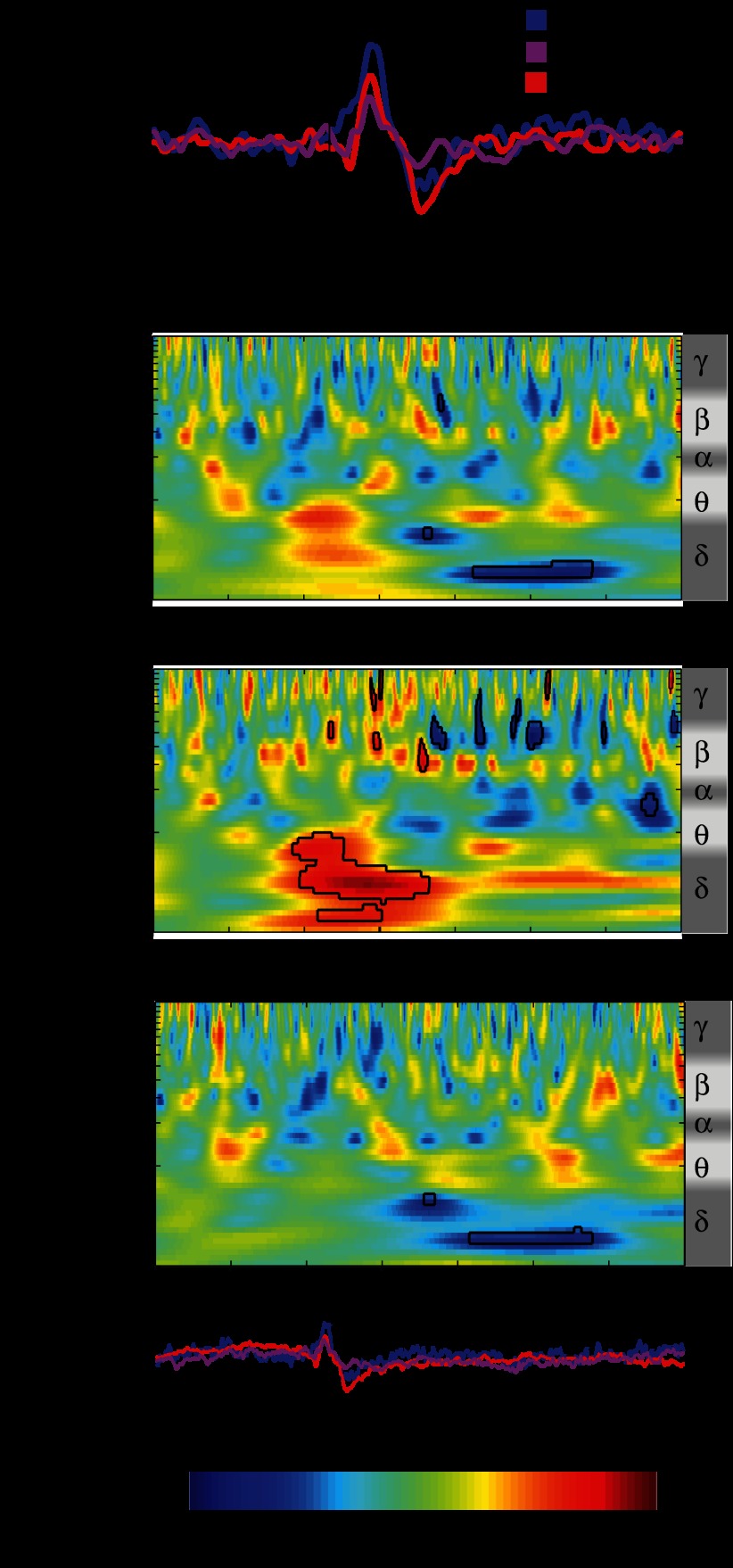
*A*: grand average ERP waveforms in response to lances fitting Aδ or C fiber-mediated pain description or neither of the 2 categories at Cz. *B*: comparison of the time-frequency decomposition between lances fitting Aδ or C fiber-mediated pain description or neither of the 2 categories at Cz. This comparison displays the difference in the phase-locked and non-phase-locked evoked activity resulting from post hoc testing. Values between 0 and 0.5 correspond to lower energy after the first term of the comparison, while values between 0.5 and 1 correspond to higher energy after the second. Circumscribed areas represent significant evoked activity differences. An approximated EEG frequency band division is displayed next to each time-frequency plot.

Finger lances described as mediated by Aδ and C fibers or by no specific fibers all evoked a clear LERP (Fig. 7*A*). Amplitude and latency were significantly different between the three categories only for P (1-way ANOVA: N amplitude *P* = 0.29; N latency *P* = 0.76; P amplitude *P* = 0.006; P latency *P* = 0.039). Post hoc testing revealed that this was due to the difference between lances described as Aδ mediated and those that did not fall into either of the two categories (unpaired *t*-test: amplitude *P* = 0.0017; latency *P* = 0.018).

The modified Bartlett's test and subsequent post hoc testing on the time-frequency decomposition showed differences within the three descriptive categories in both the late and the ultralate event-related activity patterns. In agreement with the ERP analysis, the late energy increase corresponding to the LERP is significantly greater after lances described as Aδ fiber mediated compared with those that did not fit either category in the delta/theta (1–5 Hz) frequency band. In addition, time-frequency analysis showed an ultralate delta (1–3 Hz) ERD associated with the lance responses described as C fiber mediated (Fig. 7*B*).

## DISCUSSION

In this study we have characterized, for the first time, the event-related brain activity associated with acute tissue-damaging painful skin lance in healthy adults. Analysis of verbal descriptors suggests that Aδ fibers are the predominant afferent nociceptive input relating to such tissue damage. We intended to model real-life pain experiences by choosing naive volunteers as participants and a familiar tissue-damaging single event as stimulus. However, our results would have been difficult to interpret without the fundamental information provided by experimental pain studies, which forms the basis of much of our understanding of this field today.

### Cortical Activation by Noxious Cutaneous Tissue Damage

Finger lances evoked a clear late NP complex. This was called the lance event-related potential (LERP) and had a P peak significantly greater in amplitude and latency than that of the NP complex evoked by sham control.

Mechanical stimuli excites a mixture of low- and high-threshold mechanoreceptors (Baumgartner et al. 2012), and the difference between innocuous and noxious evoked activity may be explained in terms of afferent fiber involvement and subsequent information processing. The N peak evoked by the lance and the sham control is comparable to that evoked by mechanical pinprick stimulation (Bromm and Scharein 1982; Iannetti et al. 2007; Yamauchi et al. 1981). As with our findings, this peak did not distinguish innocuous from noxious stimulation and is therefore likely to arise from Aβ tactile afferent input. On the other hand, the P peak following the lance is comparable to the positive deflection identified by Bromm and Scharein (1982), which was accompanied by pain report, and may be explained by the recruitment of Aδ nociceptors in addition to Aβ tactile afferents. However, it may also represent a difference in the saliency between an innocuous control and a noxious stimulation (Iannetti and Mouraux 2010; Legrain et al. 2011). The vertex potential following mechanical stimulation found in the present study and reported by others (Bromm and Scharein 1982; Iannetti et al. 2007; Yamauchi et al. 1981) has a shorter latency (80–280 ms) than that following laser stimulation (200–380 ms; Cruccu et al. 2008; Treede et al. 2003) but is nevertheless consistent with Aδ activation. Contact heat- and laser-evoked potentials are mediated by thermonociceptive cutaneous fibers, and it is not surprising that the mechanonociceptive evoked responses reported have a shorter latency. The skin lance is most likely to activate type I Aδ nociceptors, which have lower mechanical thresholds, slower heat responses, and faster conduction velocities, compared with type II Aδ nociceptors, which are relatively insensitive to mechanical stimulation and respond more rapidly to heat (type I mean 25 m/s compared with type II mean 14 m/s) (Treede et al. 1998). In addition, skin lance will evoke a highly synchronized volley, free from the delays involved in thermal absorption and intraepidermal nociceptor distribution.

The finger lance evoked two late regions of phase-locked energy increase in the delta/theta and alpha/beta bands occurring before 500 ms and 250 ms, respectively, and a period of ultralate beta/gamma ERS and delta/theta ERD starting after 500 ms and lasting until 2,500 ms. The regions of phase-locked energy increase after finger lance are likely to represent the LERP, and paired comparison with sham control stimulation highlights activity associated with nociceptive input or associated saliency in the delta and alpha bands (Iannetti et al. 2008), similar to those associated with late LEPs (Mouraux et al. 2003; Mouraux and Iannetti 2008). In particular the late alpha band energy increase is similar to the “late ERS” described by Mouraux et al. (2003), which was found to have a non-phase-locked and a phase-locked component. In the case of finger lance, this is mostly phase-locked and may indicate *1*) repeated stimulation inducing a latency jitter that is not present in single stimulus trials, as in the present study, and *2*) a more regulated pattern of brain activity following a tissue-damaging procedure compared with laser stimulation. The ultralate beta/gamma ERS after finger lance is not consistently greater compared with sham control stimulation and is therefore likely to be related to nonspecific aspects of the sensory input (Boiten et al. 1992; Dujardin et al. 1993; Gaetz and Cheyne 2006; Pfurtscheller and Lopes da Silva 1999). A notable finding in this study is the stronger theta/delta ERD following finger lance compared with sham control stimulation. This may reflect brain activities related to attentional and mnemonic processes required by the task of pain scoring (Iannetti et al. 2008; Mouraux et al. 2003).

The sham control stimulation evoked a clear response in only 7 of 19 single trials and only by a clinically trained clinical physiologist identifying the peak and troughs that most resembled the N and P components of the group average. Data mining techniques such as ICA and wavelet analysis to enhance the signal-to-noise ratio in single trials could have been beneficial (Hu et al. 2010). Nevertheless, our conclusions are based on group analyses, and the variability caused by the partially subjective peak selection can be considered part of the distribution variance.

### Effect of Peripheral Sensitization

Mustard oil was used in this study to test the effect of nociceptor sensitization on the LERP. Topical application of mustard oil (allyl isothiocyanate) to the skin activates underlying sensory nerve endings, producing pain, inflammation, and robust hypersensitivity to thermal and mechanical stimuli. The cellular and molecular target for the pungent action of mustard oil is TRPA1 (Jordt et al. 2004). TRPA1 is generally expressed in small- to medium-diameter, peptide-containing neurons that express the related TRPV1, a multimodal channel activated by capsaicin, heat, and inflammatory chemicals. The expression distribution depends on the target tissue, and recent estimates suggest that ∼6% of cutaneous nociceptors are TRPA1+ (Malin et al. 2011). It is also expressed by many large-caliber axons and low-threshold mechanoreceptor endings, epidermal and hair follicle keratinocytes (Kwan et al. 2009). In the present study, mustard oil increased the pain report to both pinprick and pressure, consistent with peripheral sensitization of TRPA1-expressing Aδ and C fiber mechanonociceptors, but that could also have arisen from a rapid central sensitization of dorsal horn neurons following TRPA1 nociceptor stimulation (Woolf and King 1990). Despite the clear reported mechanical sensitization, consistent with previous reports (Koltzenburg et al. 1992), mustard oil did not affect pain ratings to finger lances. This may be because the lance stimulus is so salient that nociceptive perception is saturated, precluding the possibility of enhanced pain, or that it stimulates deep nociceptors, unaffected by the topical mustard oil (Simone and Ochoa 1991).

Mustard oil also did not increase the LERP, but rather the overall energy was reduced and there was no significant difference in evoked activity in the ultralate time frame. Mixed effects on thermonociceptive evoked potentials have been reported after application of another sensitizing agent, topical capsaicin, which sensitizes the TRPV1 channel. Doses that produce heat hyperalgesia and allodynia either do not change or reduce laser-evoked pain, together with unchanged or reduced LEP amplitudes and longer latencies (de Tommaso et al. 2005; Domnick et al. 2009; Valeriani et al. 2003). Others report capsaicin increasing LEP amplitudes related to C fiber activity but not Aδ activity (Tzabazis et al. 2011), while contact heat-evoked potentials have decreased N2/P2 latencies but unchanged amplitudes after capsaicin (Madsen et al. 2012). Taken together these results suggest a central interaction of activity evoked by Aδ and C fibers, and our data indicate that enhanced C nociceptor ongoing activity following mustard oil sensitization desynchronizes the Aδ-mediated signaling (Tran et al. 2008; Truini et al. 2007).

### Nociceptive Fiber Contribution to LERP

A recent screening test involving a three-item verbal rating using the words “pricking,” “dull,” and “pressing” characterizes pain sensations evoked by a physical stimulus as transmitted via Aδ or C fibers (Beissner et al. 2010). This test was easy to administer and time efficient and provided specific and relevant information about whether the finger lance excited predominantly Aδ or C fibers in our volunteers. A wide variety of words from the McGill Pain Questionnaire were chosen to describe each lance event; however, it was clear that most descriptors were from the Aδ-fiber input range. Of the 38 lance events accepted for analysis, 20 were classified as Aδ fiber related and 4 as C fiber related and 14 could not be categorized, according to the discrimination proposed by Beissner et al. (2010). These classifications were not related to the presence of mustard oil or to the reported pain intensity.

While all lances evoked a clear NP complex, a significant difference in the latency and amplitude of the P component was found between lances described as Aδ fiber mediated and those that did not fall into any category, suggesting that the P component is related to the processing of Aδ-fiber input. In the frequency domain, this corresponds to the phase-locked energy increase in the delta/theta band. On the other hand, the late alpha/beta band ERS does not correlate with fiber type and may be more closely related to the saliency of the stimulus. The LERPs that were described as mediated by C fibers were not differing from Aδ but were followed by a significantly stronger ultralate delta ERD, suggesting that this ERD is associated with processing of C-fiber input. It is likely that any given lance is mediated by an Aβ-, Aδ-, and C-fiber input but the balance of activation differs between individuals and this is reflected in the pattern of non-phase-locked neural activity in the cortex. The three-word verbal discriminator is a convenient and noninvasive indication of peripheral input; however, it is not a direct measure of afferent fiber activation, and our interpretation requires further experimental evidence (Beissner et al. 2010).

### Methodological Considerations

Typically, experiments exploring cortical responses to sensory stimuli involve multiple repetitions of the same stimulation (in terms, for example, of modality and intensity) on the same subject. Responses for each subject/condition can then be obtained by averaging single sweeps, allowing higher signal-to-noise ratio and group analyses on these averaged trials. As a result, each group may contain data from, for example, 20 trials × 10 subjects = 200 trials. In the present study, where frank tissue damage occurred, we were limited to recording single responses from 20 subjects, therefore not permitting analysis on an individual basis. Nevertheless, the group analysis allowed us to identify statistically significant patterns (above the noise/variance level) characteristic of the EEG response to a skin-breaking procedure.

Ideally, to avoid bias due to stimulus anticipation, control stimulation and lance should be applied in a pseudorandom order. However, because of the tissue damage caused by the lance, this was not possible. Therefore the pain expectancy could have been higher before the lance than the sham control. However, this has been shown not to affect the vertex potential evoked by noxious laser stimulations (Brown et al. 2008; Lorenz et al. 2005). Although caution was taken to blind the participants to the side of mustard oil application, the burning sensation caused by the compound was unavoidable. The sensitization of TRPA+ C fibers, however, is a peripheral effect that does not depend on subjective awareness, and this was the purpose of this treatment rather than the response to mustard oil per se. Nevertheless, the unblinding problem in testing irritant compounds such as mustard oil or capsaicin is well known, and currently there are no entirely satisfactory solutions to it (Gooding et al. 2010).

### Conclusions

Noxious cutaneous tissue damage causes a distinct late ERP (lance event related potential, LERP) that can be measured at the vertex with a latency of 100–300 ms that is likely to reflect Aβ and Aδ fiber-evoked somatosensory cortical processing modulated by saliency. This LERP also consists of a phase-locked energy increase between 1 and 20 Hz, which can be disrupted by ongoing activity from TRPA1+ C fibers, supporting the idea of central interaction of Aδ- and C-fiber inputs. The use of a verbal descriptor screening test suggested that lance pain experience is predominantly due to Aδ fiber activation, which results in the late LERP, and that, when individuals describe lances as C fiber mediated, an ultralate delta band ERD follows this response. Thus we conclude that pain evoked by acute tissue damage is associated with distinct Aδ and C fiber-mediated patterns of synchronization and desynchronization of EEG oscillations in the brain. Lances are routinely used for blood sampling, especially in neonatal intensive care, where they are known to cause a clear nociceptive response in the cortex (Fabrizi et al. 2011; Slater et al. 2010). The results and analysis presented here could therefore be used to map and interpret the development of Aδ and C fiber-mediated patterns of EEG synchronization and desynchronization in the newborn brain.

## GRANTS

This work was supported by the Wellcome Trust. S. Olhede was supported by the Engineering and Physical Sciences Research Council.

## DISCLOSURES

No conflicts of interest, financial or otherwise, are declared by the author(s).

## AUTHOR CONTRIBUTIONS

Author contributions: L.F., G.W., R.S., and M.F. conception and design of research; L.F., A.L., and S.O. analyzed data; L.F., G.W., J.M., and M.F. interpreted results of experiments; L.F. and G.W. prepared figures; L.F. and G.W. drafted manuscript; L.F., G.W., A.L., J.M., S.O., and M.F. edited and revised manuscript; L.F., G.W., A.L., J.M., R.S., S.O., and M.F. approved final version of manuscript; G.W. and A.L. performed experiments.

## APPENDIX: TF-SINGLE AND TF-GROUP MODELING

The estimation of the time-frequency spectral energy content of the EEG signal was conducted in two ways: *1*) by calculating the energy of the TF transform for each individual trial and then averaging them in the time-frequency domain (TF-single) (Mouraux et al. 2003) and *2*) by averaging the trials in the time domain and then calculating the energy of the TF transform of the resulting average (TF-group). To assess the significance of the energy changes induced by the different stimuli compared with ongoing background EEG and to confront the patterns of evoked activity between different groups, we specified the normalized estimators for TF-single and TF-group and their modeling distributions.

Individual trials were grouped according to the stimulation type and the compound present on the stimulated finger into *1*) lances on fingers treated with inactive compound (LIC), *2*) lances on fingers treated with mustard oil (LMO), and *3*) sham controls (SC). Subsequently, all lance trials were also grouped according to verbal description into events that fitted the Aδ fiber (LAδ)- or C fiber (LC)-mediated pain definition or did not fit in either of these two classifications (LNo).

We started by defining a model for the observed EEG signal assuming that this signal is a superimposition of effects due to the ongoing background EEG activity and the activity evoked by the stimulation. The observed EEG signal *X*^(*kr*)^(*t*) for trial/subject *k* (with *k* = 1, …, *N* where *N* is the number of trials/subjects) from a given group *r* (with *r* = LIC, LMO, or SC) can then be described as

(1) $$X^{\left(kr\right)}\left(t\right)=X_{\text{n}}^{\left(kr\right)}\left(t\right)+X_{\text{s}}^{\left(kr\right)}\left(t\right)$$

where *X*_s_^(*kr*)^(*t*) is the signal we wish to extract and *X*_n_^(*kr*)^(*t*) is the ongoing background EEG (i.e., noise). Time-frequency analysis was performed with a complex Morse wavelet transform (Olhede and Walden 2002), and because this is a linear operation, it results that

(2) $$W^{\left(kr\right)}\left(a,b\right)=W_{\text{n}}^{\left(kr\right)}\left(a,b\right)+W_{\text{s}}^{\left(kr\right)}\left(a,b\right)$$

where *W*_n_^(*kr*)^(*a*,*b*) is the wavelet transform of the noise *X*_n_^(*kr*)^(*t*) and *W*_s_^(*kr*)^(*a*,*b*) is the wavelet transform of the signal *X*_s_^(*kr*)^(*t*). Moreover, considering *X*_n_^(*kr*)^(*t*) as a Gaussian process (e.g., the realization for any set of time points being jointly normal), it follows that at any given time point *b* the wavelet transform has distribution

(3) $$W_{\text{n}}^{\left(kr\right)}\left(a,b\right)∼N_{C}\left(0,\sigma_{r}^{2}\left(a\right)\right)$$

while the signal of interest has a more heterogeneous temporal structure,

(4) $$W_{\text{s}}^{\left(kr\right)}\left(a,b\right)∼N_{C}\left(0,\sigma_{r}^{2}\left(a,b\right)\right)$$

To assess the significance of the energy changes represented by TF-single for a given group *r* we compared the sample mean energy (i.e., modulus square) *S̃*^(*r*)^(*a*,*b*)—obtained by averaging of the *N* wavelet transforms of the individual trials/subjects *k* belonging to that group—against the noise σ_*r*_^2^(*a*). This is equivalent to defining a normalized estimator *S̃*^(*r*)^(*a*,*b*) of TF-single as

(5) $${\tilde{S}}^{\left(r\right)}\left(a,b\right)=\frac{{\hat{S}}^{\left(r\right)}\left(a,b\right)}{\sigma_{r}^{2}\left(a\right)}$$

where (sample averaging)

(6) $${\hat{S}}^{\left(r\right)}\left(a,b\right)=\frac{1}{N}\sum_{k=1}^{N}{\left|W^{\left(kr\right)}\left(a,b\right)\right|}^{2}$$

and σ_*r*_^2^(*a*) can be estimated from the baseline period *T* preceding the stimulation as

(7) $${\hat{\sigma }}_{r}^{2}\left(a\right)=\frac{1}{\left|T\right|}\sum_{b\in T}{\hat{S}}^{\left(r\right)}\left(a,b\right)$$

To conduct an appropriate statistical test on the normalized estimator described in *Eq. 5*, we need to specify a suitable modeling distribution. From *Eq. 3* we have that

(8) $$\frac{{\left|W^{\left(kr\right)}\left(a,b\right)\right|}^{2}}{\sigma_{r}^{2}\left(a\right)}∼\frac{1}{2}\chi_{2}^{2}\;\forall \;k$$

and therefore, considering *Eqs. 5* and *6*, that

(9) $${\tilde{S}}^{\left(r\right)}\left(a,b\right)∼\frac{1}{2N}\chi_{2N}^{2}$$

The statistical significance of the energy changes represented by TF-single and modeled by *S̃*^(*r*)^(*a*,*b*) can then be tested with a two-tailed χ^2^-test.

Now we intend to specify a similar model and test for TF-group. To do this for a given group *r*, we compared the energy ${\hat{\bar{S}}}^{\left(r\right)}\left(a,b\right)$ of the mean EEG trace of the sample—obtained by averaging of the *N* individual trials/subjects *k* belonging to that group—against the noise σ_*r*_^2^(*a*). Starting again from *Eqs. 3* and *4*, this is equivalent to defining a normalized estimator ${\hat{\bar{S}}}^{\left(r\right)}\left(a,b\right)$ of TF-group as

(10) $${\tilde{\bar{S}}}^{\left(r\right)}\left(a,b\right)=\frac{{\hat{\bar{S}}}^{\left(r\right)}\left(a,b\right)}{\sigma_{r}^{2}\left(a\right)}$$

where, considering that the Morse wavelet transformation is a linear operation,

(11) $${\tilde{\bar{S}}}^{\left(r\right)}\left(a,b\right)={\left|\frac{1}{N}\sum_{k=1}^{N}W^{\left(kr\right)}\left(a,b\right)\right|}^{2}={\left|{\bar{W}}^{\left(r\right)}\left(a,b\right)\right|}^{2}$$

and σ_*r*_^2^(*a*) can be estimated as in *Eq. 7*. Assuming *Eqs. 3* and *4*, it results that the sample mean of the wavelet transform *W̄̄*^(*r*)^(*a*,*b*) is distributed as

(12) $${\bar{W}}^{\left(r\right)}\left(a,b\right)∼N_{C}\left(0,\frac{\sigma_{r}^{2}\left(a\right)}{N}\right)$$

and therefore the modeling distribution for the normalized estimator of TF-group in *Eq. 10* can be specified as

(13) $${\tilde{\bar{S}}}^{\left(r\right)}\left(a,b\right)∼\frac{1}{2N}\chi_{2}^{2}$$

The statistical significance of the energy changes represented by TF-group and modeled by ${\tilde{\bar{S}}}^{\left(r\right)}\left(a,b\right)$ can then be tested with a two-tailed χ^2^-test.

To compare the patterns of evoked activity between different conditions we conducted a within-subject pairwise comparison. The TFs-single were compared between lances and sham controls (LIC vs. SC) and between lances on fingers treated with inactive compound or mustard oil (LIC vs. LMO). Considering *N* pairs of trials *k* from the same subject in conditions *r*_1_ and *r*_2_ (i.e., LIC vs. SC or LIC vs. LMO), a normalized estimator of the within-subject pairwise difference in TFs-single can be defined as

(14) $${\tilde{D}}^{\left(r_{12}\right)}\left(a,b\right)=\frac{{\hat{D}}^{\left(r_{12}\right)}\left(a,b\right)}{\sigma_{r_{12}}^{2}\left(a\right)}$$

where

(15) $${\hat{D}}^{\left(r_{12}\right)}\left(a,b\right)=\frac{1}{N}\sum_{k=1}^{N}\left({\left|W^{\left(kr_{1}\right)}\left(a,b\right)\right|}^{2}-{\left|W^{\left(kr_{2}\right)}\left(a,b\right)\right|}^{2}\right)$$

and σ_*r*_12__^2^(*a*) can be estimated as in *Eq. 7*. To specify the appropriate modeling distribution for ${\tilde{D}}^{\left(r_{12}\right)}\left(a,b\right)$, we start by defining the difference in TF-single between conditions *r*_1_ and *r*_2_ for a given subject *k* as

(16) $$Z^{\left(kr\right)}\left(a,b\right)=\left({\left|W^{\left(kr_{1}\right)}\left(a,b\right)\right|}^{2}-{\left|W^{\left(kr_{2}\right)}\left(a,b\right)\right|}^{2}\right)\;\forall \;k$$

From *Eq. 8* we get that

(17) $$Z^{\left(kr\right)}\left(a,b\right)∼\frac{1}{2}\chi_{2}^{2}\sigma_{r}^{2}\left(a\right)-\frac{1}{2}\chi_{2}^{2}\sigma_{r}^{2}\left(a\right)∼\text{Exp}\left(1\right)-\text{Exp}\left(1\right)$$

where Exp(1) is the exponential distribution with mean 1. Therefore

(18) $$\frac{Z^{\left(kr\right)}\left(a,b\right)}{\sigma_{r}^{2}\left(a\right)}∼\text{Laplace}\left(0,1\right)$$

and, according to the central limit theorem,

(19) $$\sum_{k=1}^{N}\frac{Z^{\left(kr\right)}\left(a,b\right)}{\sigma_{r}^{2}\left(a\right)}∼N\left(0,2N\right)$$

A two-tailed *z*-test could then be conducted on

(20) $${\tilde{D}}^{\left(r_{12}\right)}\left(a,b\right)∼N\left(0,\frac{2}{N}\right)$$

Lances were then regrouped according to verbal description into lances described as mediated by Aδ (LAδ), C (LC), or no specific (LNo) fibers. To assess whether there was a difference in the patterns of evoked activity between these categories we compared their TFs-single with a modified Bartlett's test (Snedecor and Cochran 1980). In case of rejection of the null hypothesis, we performed a post hoc analysis to assess which categories were significantly different from each other, but only in the time-frequency regions that were found significantly different in the modified Bartlett's test. To compare the TF-single associated to category *c*_1_ with the TF-single associated to category *c*_2_ we looked at the ratio between these two quantities:

(21) $$T^{\left(c_{12}\right)}=\frac{{\tilde{S}}^{\left(c_{1}\right)}\left(a,b\right)}{{\tilde{S}}^{\left(c_{2}\right)}\left(a,b\right)}∼\frac{\frac{1}{2N}\chi_{2N}^{2}}{\frac{1}{2N}\chi_{2N}^{2}}∼F_{2N,2N}$$

where the categories *c*_1_ and *c*_2_ represent LAδ, LC, or LNo. The statistical significance of this quantity could then be tested with a two-tailed *F*-test.
